# Atlas of Legal Medicine

**Published:** 1899-02

**Authors:** 


ATLAS OF LEGAL MEDICINE.—By Dr. E. Von Hoffman, Professor of Legal Medicine and Director of the Medico-Legal Institute at Vienna. Authorized Translation from the German. Edited by Frederick Peterson, M. D., Clinical Professor of Mental Diseases in the Woman's Medical College, New York, etc., assisted by Aloysius O. J. Kelley, M. D., Instructor in Physical Diagnosis, University of Pennsylvania, etc., etc. Fifty-six plates in colors and 193 illustrations in black. Published by W. B. Saunders, Philadelphia. 1898. Price, cloth, $3.50 net.

This is another of this beautiful series of hand atlases, and fully sustains the high character of the preceding volumes. I almost wondered when I first saw the title how it was possible to discuss the subject of legal medicine in an atlas. While, of course, it is impossible to go into all the details by this method, yet it is surprising how much valuable technical information is conveyed by these magnificent colored plates and illustrations, and the short descriptive text accompanying them. In fact, the scope of the work is as ample as that of the best text-books of the subject, and indeed it is intended to accompany and supplement the text of such works by illustrating them. I. J. J.

